# Retrospective Study of Phospholipase A2 Receptor and IgG Subclasses in Glomerular Deposits in Chinese Patients with Membranous Nephropathy

**DOI:** 10.1371/journal.pone.0156263

**Published:** 2016-05-25

**Authors:** Hong-rui Dong, Yan-yan Wang, Xiao-hong Cheng, Guo-qing Wang, Li-jun Sun, Hong Cheng, Yi-pu Chen

**Affiliations:** 1 Division of Nephrology, Beijing Anzhen Hospital, Capital Medical University, Beijing, P.R. China; 2 Division of Nephrology, Shanxi Traditional Chinese Medicine Hospital, Xian, P.R. China; Postgraduate Medical Institute, INDIA

## Abstract

**Background and objectives:**

The research work in the past years showed that detection of phospholipase A2 receptor (PLA2R) antigen and its dominant IgG4 autoantibody in glomerular deposits of patients with membranous nephropathy (MN) was useful for the differentiation between primary MN (PMN) and secondary MN (SMN), but so far such research data from large Chinese patient series is little. Here, we are going to report a research work in a Chinese cohort.

**Design, setting, participants, & measurements:**

This study enrolled 179 patients with PMN, 40 patients with membranous lupus nephritis (LN-MN), 26 patients with hepatitis B virus-associated MN (HBV-MN), 2 patients with malignancy-associated MN (M-MN) and one patient with IgG4-related MN (IgG4-MN). PLA2R and IgG subclasses in glomerular deposits of these patients were examined by immunofluorescence and/or immunohistochemical staining, and the potential value of the above examinations for differential diagnosis of PMN and SMN was evaluated.

**Results:**

Glomerular PLA2R deposition was present in 92.2% patients with PMN and 7.7% patients with HBV-MN, but none of the patients with LN-MN. Predominant/codominant IgG4 deposition was found in 93.3% patients with PMN and 11.5% patients with HBV-MN, but none of the patients with LN-MN. The two M-MN patients both had glomerular PLA2R and predominant/codominant IgG4 deposition. The one IgG4-MN patient had deeply staining IgG4 but no PLA2R in glomeruli.

**Conclusions:**

The glomerular PLA2R and predominant/codominant IgG4 deposition is frequently observed in Chinese patients with PMN. Immunofluorescence and immunohistochemical staining of renal biopsy tissue for detection of glomerular PLA2R and IgG subclasses deposition can help to distinguish PMN from LN-MN and most of HBV-MN.

## Introduction

Membranous nephropathy (MN) is a common pathological pattern of glomerular diseases, accounting for about 20% of the nephrotic syndrome in adults [1]. Among the adult patients undergoing renal biopsy in our division, MN accounts for 22.5% which is second only to IgA nephropathy. MN is characterized by the deposition of immune complexes in the subepithelial space and the diffuse thickening of the glomerular basement membrane [1–3]. Approximately 75–80% of all MN is primary membranous nephropathy (PMN), also referred to as idiopathic MN, which occurs in the absence of any identifiable cause or inciting event; the remainder is secondary membranous nephropathy (SMN), which is caused by an systemic disease or a well-recognized etiologic factor, such as systemic lupus erythematosus (SLE), hepatitis B virus (HBV) infection or malignancy [1–4].

Current studies indicate that PMN is an autoimmune disease [1–5]. The M-type phospholipase A2 receptor (PLA2R) on cell surface of podocytes is the major autoantigen in most patients with PMN [1–5]. In SMN, however, the immune reaction is caused by non-renal autoantigens or exogenous antigens [1–4]. It has been known that the autoantibody of PLA2R is predominantly IgG4 subclass, which is often accompanied by other IgG subclasses in smaller amounts [1–4,6]. In contrast, the antibodies in patients with SMN are predominantly other IgG subclasses rather than IgG4 [1,6]. Therefore, testing serum PLA2R antibody and detecting PLA2R and IgG subclasses in glomerular deposits might help to distinguish between PMN and SMN.

Serum PLA2R antibodies in adult patients with MN have been widely studied by many nephrologists in the North American and European countries[7–16], and the Asian countries including China [17–21], but the data of PLA2R staining, especially accompanied with IgG subclasses staining, in renal biopsy tissues of large series of adult MN patients are relatively limited [13–17,22]. In this study, the glomerular deposits of PLA2R and IgG subclasses in a large series of Chinese patients with PMN and SMN were retrospectively studied by using immunofluorescence and immunohistochemical staining, and the potential value of these examinations for differential diagnosis of PMN and SMN was explored.

## Materials and Methods

### Patients and diagnostic criteria

The study was approved by the Ethics Review Committee of Beijing Anzhen Hospital, Capital Medical University and implemented in accordance with the Declaration of Helsinki. The members enrolled in this study all signed a written informed consent form.

The diagnosis of MN in this study was relied on pathological examinations including immunofluorescence, light and electron microscopy of renal biopsy tissue [1,2]. PMN was diagnosed after exclusion of secondary causes such as autoimmune diseases (e.g., SLE, rheumatoid arthritis, Sjogren syndrome), infection (e.g., hepatitis B, hepatitis C, syphilis), malignancies (e.g., colon cancer, lung cancer, lymphoma), drugs (e.g., nonsteroidal anti-inflammatory drugs, penicillamine) and toxicants(e.g., mercury) [1–4]. SLE and membranous lupus nephritis (LN-MN) were diagnosed according to the classification criteria revised by the Systemic Lupus International Collaborating Clinics (SLICC) in 2012 and the classification criteria revised by the International Society of Nephrology and Renal Pathology Society (ISN/RPS) in 2003, respectively [23,24]. HBV-associated MN (HBV-MN) was diagnosed by the following criteria: (i) persistent HBV antigenemia, (ii) finding at least one HBV antigen in glomerular capillary wall, and (iii) no other secondary causes of MN [25,26]. The reference criteria for diagnosis of malignancy-associated MN (M-MN) were as follows: (i) malignancy identified at the time of renal biopsy or within one year after diagnosis of MN, and (ii) no other findable secondary causes of MN [22,27,28]. IgG4-related MN (IgG4-MN) was diagnosed by the diagnostic criteria for IgG4-related kidney disease established by Japanese Society of Nephrology in 2011 [29], and it was often accompanied by IgG4-related tubulointerstitial nephritis [30]. The original data of the study see S1 Table.

### Detection of PLA2R in glomerular deposits by immunohistochemical staining

Paraffin-embedded kidney tissues were cut into 4μm-thick sections, and then processed by autoclave heating-induced epitope retrieval (in citrate solution, pH 6.0). Rabbit anti-human PLA2R polyclonal antibody (Sigma, 1:800 dilution) was used as primary antibody and alkaline phosphatase (ALP) labeled goat anti-rabbit IgG antibody (Beijing Zhongshan) was used as secondary antibody. ALP-Red was used as substrate of ALP. Nucleus was stained with hematoxylin. Renal biopsy tissues of 5 patients with minimal change disease were also stained as negative controls.

In normal kidney tissue there is very weak expression of PLA2R on the glomerular podocytes [13,14] and it was recognized as negative result in this study. When strong PLA2R staining distributed along the glomerular capillary wall in a fine granular pattern, the result was judged as positive.

### Detection of IgG subclasses in glomerular deposits by immunofluorescence assay

Frozen kidney tissues were cut into sections of 5μm-thick, and then used for direct immunofluorescence staining by using fluorescein isothiocyanate (FITC)-labeled monoclonal antibody against each IgG subclass (Sigma, 1:50 dilution). The shape, location and fluorescence intensity of glomerular deposits were analyzed.

### Detection of HBsAg and HBcAg in glomerular deposits by immunofluorescence and immunohistochemical staining

**Fig 1:**
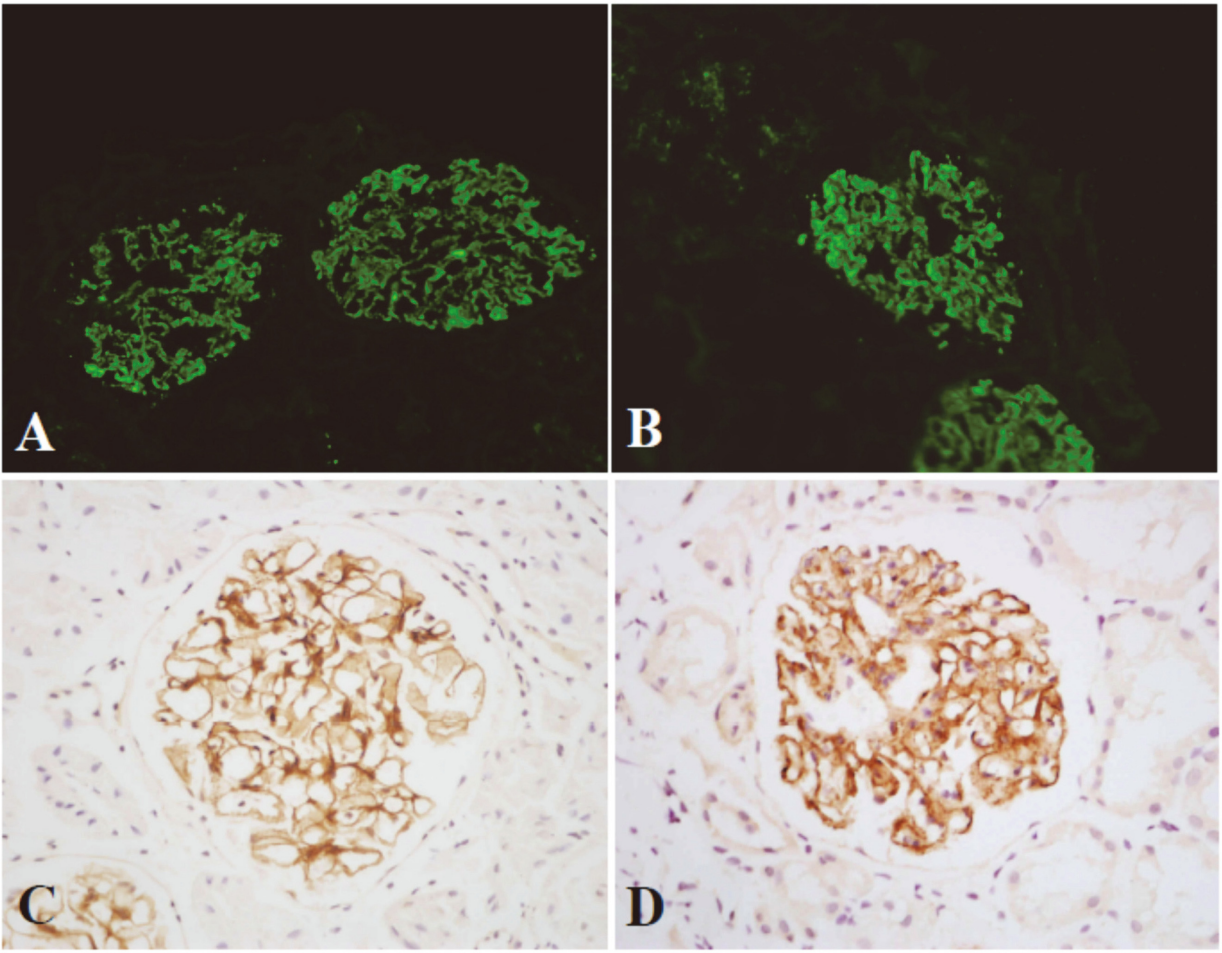
Immunofluorescence and immunohistochemical staining of HBsAg and HBcAg in glomerular deposits. HBsAg (A and C) and HBcAg (B and D) are distributed along the glomerular capillary walls in a fine granular pattern. immunofluorescence (A and B, ×200) and immunohistochemical assays (C and D, HRP×400).

The granular deposition of HBV antigen(s) on glomerular capillary wall is a key evidence for diagnosis of HBV-MN. To obtain reliable results, HBsAg and HBcAg in glomerular deposits in this study were detected by both immunofluorescence and immunohistochemical assays. According to our rule, only the above two kinds of staining are simultaneously positive, it can be judged as HBsAg or/and HBcAg deposition in glomeruli (Fig 1).

For indirect immunofluorescence assay of HBsAg and HBcAg, frozen kidney tissues were cut into 5μm-thick sections. Mouse anti-human HBsAg monoclonal antibody (DAKO, 1:100 dilution) and rabbit anti-human HBcAg polyclonal antibody (DAKO, 1:100 dilution) were used as primary antibodies. FITC-labeled rabbit anti-mouse immunoglobulins and FITC-labeled swine anti-rabbit immunoglobulins were used as secondary antibodies, respectively.

For immunohistochemical staining of HBsAg and HBcAg, paraffin-embedded kidney tissues were cut into 4μm-thick sections and then processed by heating-induced and trypsin-induced double epitope retrieval. Mouse anti-human HBsAg monoclonal antibody (Beijing Zhongshan, Clone ZCH16) and rabbit anti-human HBcAg polyclonal antibody (Beijing Zhongshan) were used as primary antibodies. Horseradish peroxidase (HRP)-labeled goat anti-mouse IgG antibody and HRP-labeled goat anti-rabbit IgG antibody were used as secondary antibodies respectively. The rest procedures were the same as mentioned above.

All results of immunofluorescence and immunohistochemical assays were evaluated by at least two renal pathologists.

### Statistical analysis

Continuous variables with normal distribution were presented as the mean ± standard deviation and comparison between groups was analyzed with one-way ANOVA. Categorical variables were described as percentage and comparison between groups was analyzed with Fisher exact test. All statistical analyses were performed with SPSS 15.0 software. The difference of P<0.05 was considered to have statistical significance.

## Results

### Clinical baseline characteristics

Total 248 consecutive patients diagnosed as MN in our Division of Nephrology from January 2012 to June 2015 were all enrolled in this study, including 179 patients with PMN, 40 with LN-MN, 26 with HBV-MN, 2 with M-MN and one with IgG4-MN. Clinical and laboratory data of patients at the time of renal biopsy are shown (Table 1). The mean age of patients with PMN was significantly older than the patients with LN-MN and HBV-MN (*P*<0.05). Besides this, the rest parameters were not statistically different. The patients with massive protein (≥ 3.5g/d) accounted for 64.1% in PMN and 43.5% in SMN. In all the 26 HBV-MN patients, the serum HBsAg was positive and 16 patients who received HBV-DNA tests all showed positive results. Before renal biopsy, 17.3% patients with PMN and 57.5% patients with LN-MN underwent corticosteroid and/or immunosuppressants treatment, but no patients with HBV-MN, M-MN and IgG4-MN received these drugs. Modified glomerular filtration rate estimating equation was used to calculate estimated glomerular filtration rate (eGFR) [31].

**Table 1:** The clinical and laboratory data.

	PMN	LN-MN	HBV-MN	M-MN	IgG4-MN
Gender (M/F)	102/77	35/5	22/4	1/1	0/1
Age (years)	49.0±14.3*	34.9±11.9	37.4±12.1	74, 58	57
Proteinuria (g/d)	5.1±3.5	4.6±3.0	3.6±2.8	8.3, 3.0	2.0
Serum albumin (g/L)	25.9±7.2	25.0±7.3	30.6±8.4	20.5, 2.1	30.0
Serum cholesterol (mmol/L)	7.7±2.6	6.9±2.5	6.6±2.5	6.6, 7.6	6.6
Serum triglyceride (mmol/L)	3.0±4.8	2.9±1.7	2.9±3.3	2.9, 2.9	4.4
Serum creatinine (μmol/L)	71.8±21.3	72.9±36.5	71.3±12.6	87.0, 55.4	52.2
eGFR (ml/min·1.73m^2^)	112.4±36.8	114.3±52.9	117.6±32.8	79.2, 103.9	111.7

Values are presented as mean±standard deviation in PMN, LN-MN and HBV-MN groups; Values are the actual test values in M-MN and IgG4-MN groups.

* *P <* 0.05 vs LN-MN and HBV-MN groups.

### PLA2R in glomerular deposits

**Fig 2:**
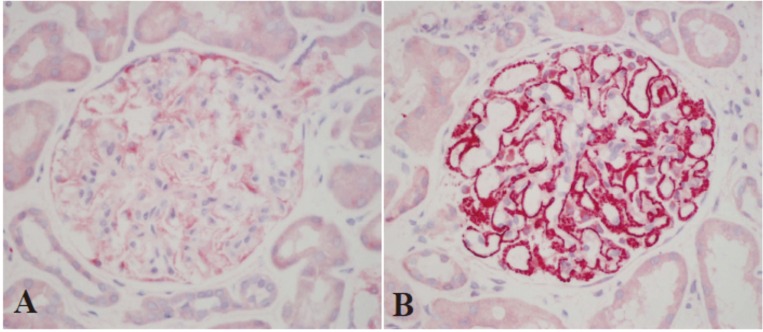
Immunohistochemical staining of PLA2R in glomerular deposits (ALP ×400). **(A)** In patients with minimal change disease there is very weak expression of PLA2R on the glomerular potocytes and it is recognized as negative result in this study. (B) In patients with PMN there is strongly stained PLA2R distributed along the glomerular capillary walls in a fine granular pattern.

Immunohistochemical staining showed PLA2R deposited along the glomerular capillary wall in a fine granular pattern in 165 (92.2%) patients with PMN and two (7.7%) patients with HBV-MN (Fig 2). Such PLA2R deposition was not found in the remaining patients with PMN or HBV-MN and all the patients with LN-MN. The two patients with M-MN both showed positive results and the one patient with IgG4-MN showed negative result. The PLA2R staining was also investigated in 5 patients with minimal change disease and all showed negative results (Fig 2).

### IgG subclasses in glomerular deposits

**Fig 3:**
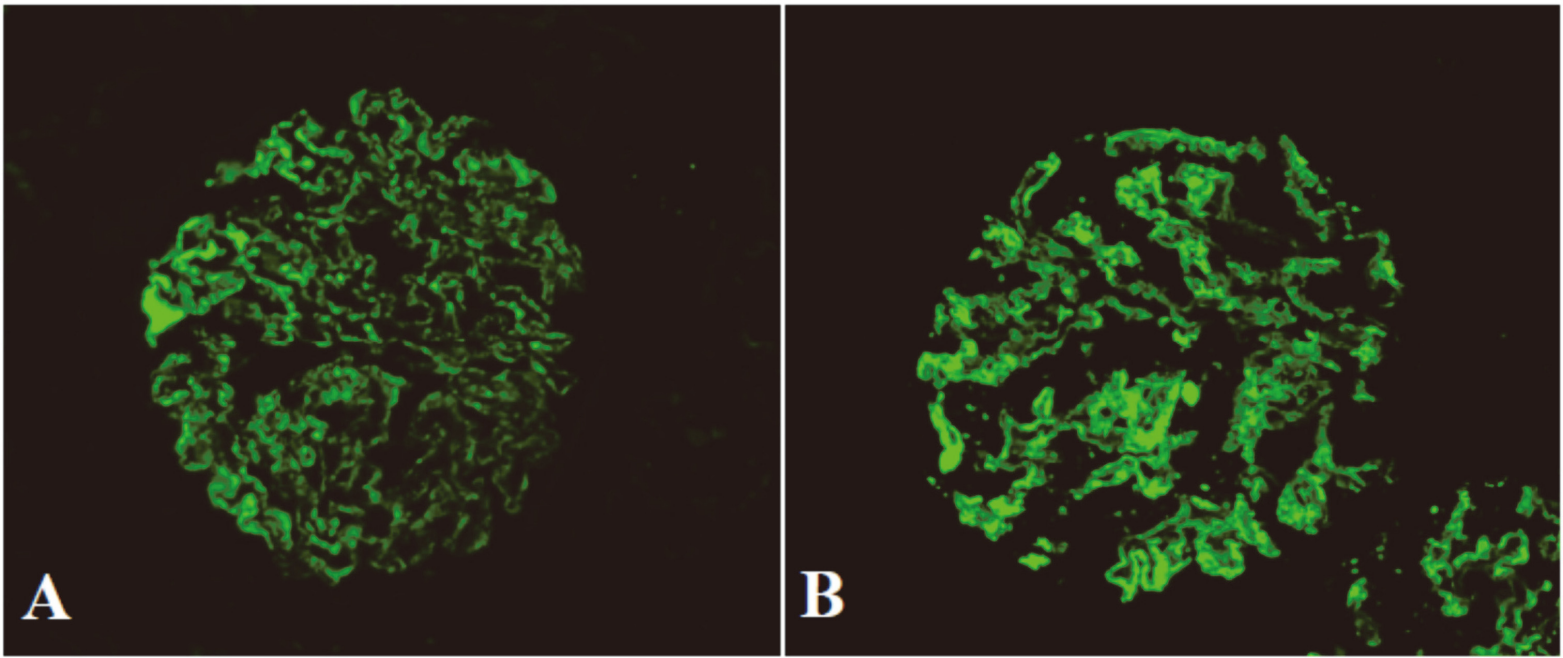
Immunofluorescence assay of IgG and IgG4 in glomerular deposits (×400). In patients with PMN IgG (A) and IgG4 (B) are distributed along the glomerular capillary walls in a fine granular pattern.

Of 179 patients with PMN, 167 (93.3%) cases had predominant/codominant glomerular IgG4 deposition (*P*<0.01) (Fig 3, Table 2), which was often accompanied with other weaker IgG subclasses (with one IgG subclass in 76 cases, two subclasses in 19 cases and three subclasses in 2 patients). The predominant/codominant IgG subclass in glomerular deposits was unchanged in different stages of PMN, and IgG subclass switching during the progression of PMN was not found (Tables 3 and 4).

**Table 2:** Predominant/codominant IgG subclasses in glomerular deposits.

IgG subclass	PMN n = 179(%)	LN-MN n = 40(%)	HBV-MN n = 26(%)	M-MN n = 2	IgG4-MN n = 1
**IgG1**	21(11.7)	9(22.5)	6(23.1)	0	0
**IgG2**	5(2.8)	27(67.5)^b^	8(30.8)	0	0
**IgG3**	4(2.2)	23(57.5)^b^	12(46.2)^c^	0	0
**IgG4**	167(93.3)^a^	0	3(11.5)	2	1

^a^*P<*0.001 vs IgG1, IgG2 and IgG3

^b^*P<*0.001 vs IgG1 and IgG4

^c^*P<*0.05 vs IgG1、IgG2 and IgG4.

**Table 3:** The percentages of predominant/codominant IgG subclass in different stages of PMN.

IgG subclass	StageI n = 56(%)	Stage I-II n = 59(%)	Stage II n = 44(%)	Stage II-III n = 15(%)	Stage III n = 5(%)
**IgG1**	7(12.5)	7(11.9)	3(6.8)	3(20.0)	1(20.0)
**IgG2**	0(0)	1(1.7)	1(2.3)	1(6.7)	2(40.0)
**IgG3**	1(1.8)	1(1.7)	1(2.3)	0(0)	1(20.0)
**IgG4**	54(96.4) *	56(94.9) *	41(93.2) *	14(93.3) *	2(40.0)

* *P*<0.01 vs IgG1, IgG2 and IgG3.

**Table 4:** The average staining intensity of IgG subclasses in different stages of PMN (mean±s.d.).

IgG subclass	Stage I n = 56	Stage I-II n = 59	Stage II n = 44	Stage II-III n = 15	Stage III n = 5
**IgG1**	0.87±0.88	0.69±0.88	0.78±0.93	0.77±1.05	0.70±0.97
**IgG2**	0.52±0.68	0.54±0.71	0.30±0.59	0.57±0.78	1.00±0.93
**IgG3**	0.45±0.72	0.65±0.80	0.39±0.63	0.33±0.70	0.60±1.34
**IgG4**	2.27±0.59*	2.45±0.57*	2.49±0.73*	2.30±0.46*	1.20±1.30

*<0.001.

In 40 patients with LN-MN, the predominant/codominant IgG subclasses in glomerular deposits were IgG2 and IgG3, which positive rates were significantly higher than other IgG subclasses (*P*<0.001) (Table 2). Weaker IgG4 deposition accompanied with dominant other IgG subclass was found in 2 patients. No predominant/codominant glomerular IgG4 deposition was observed.

In 26 patients with HBV-MN, the predominant/codominant IgG subclass in glomeruli was IgG3, which positive rate was significantly higher than other IgG subclasses (*P*<0.05) (Table 2). However, predominant/codominant glomerular IgG4 deposition was still found in 3 (11.5%) patients (Table 2).

In addition, in 2 patients with M-MN, one had stronger IgG4 and another had stronger IgG4 with weaker IgG3 in glomerular deposits. In the only one patient with IgG4-MN there was deeply staining IgG4 on glomerular capillary wall (Table 2).

### The relationship between PLA2R and predominant/codominant IgG4 in glomerular deposits

Of 179 patients with PMN, 158 (88.3%) patients simultaneously had PLA2R and predominant/codominant IgG4 deposition, 9 (5.0%) had predominant/codominant IgG4 but no PLA2R deposition, and 7 (3.9%) had PLA2R but no predominant/ codominant IgG4 deposition. Both PLA2R and IgG4 were negative in 5 (2.8%) patients. Of 40 patients with LN-MN, no one had PLA2R and predominant/ codominant IgG4 deposition. Of 26 patients with HBV-MN, 2 (7.7%) patients had both PLA2R and predominant/codominant IgG4 deposition, and one (3.8%) had predominant/codominant IgG4 but no PLA2R deposition. Two M-MN patients had PLA2R as well as predominant IgG4 deposition. The only one IgG4-MN patient had predominant IgG4 but no PLA2R deposition.

## Discussion

PMN is the most common pathological pattern in adult nephrotic syndrome, and usually considered as a glomerular podocyte disease caused by autoimmune reaction [1–5]. Through efforts of decades, until recent years two kinds of key antigens located on the podocyte surface are identified, which are involved in the pathogenesis of adult PMN [32,33]. One is PLA2R which was discovered by Beck and colleagues in 2009 [5], and another is thrombospondin type-1 domain-containing 7A (THSD7A) which was first reported by Tomas and colleagues in 2014 [34]. Current studies reveal that PLA2R is the major antigen which induces the pathogenic autoantibodies in most patients with PMN [1–5]. Circulating anti-PLA2R autoantibody was present in approximately 52.0–77.8% patients with PMN in North American and European countries [7–16], about 66.7% in India and 53.0–69.0% in Japan and Korea [17–19, 35]. PLA2R antigen in glomerular deposits was found in approximately 69.2–76.6% patients with PMN in North American and European countries [13–16, 22], 75% in India and 63.6% in Japan [17,35]. Data from China showed 81.7% patients with PMN had circulating anti-PLA2R autoantibody [21], now our study displays 92.2% patients with PMN had glomerular PLA2R deposition. Is the proportion of PLA2R-related cases in Chinese patients with PMN higher than that in above countries? It should be concerned, but the conclusion cannot be drawn at the present time because of the insufficient research samples. For clarifying this problem, multicenter clinical trials with large sample are needed.

The autoantibody of PLA2R is predominantly IgG4 subclass [1–4], which had been found coexisting with PLA2R in glomerular deposits of PMN patients [5,36]. In the present study, among 179 patients with PMN, 158 showed both predominant/ codominant IgG4 and PLA2R deposition in glomeruli. In the remaining 21 patients, 9 showed predominant/codominant IgG4 but no PLA2R, and 7 showed PLA2R but no predominant/codominant IgG4 deposition in glomeruli. The former situation was also observed by other investigators in adult and pediatric PMN patients [15,22,37]. One possible explanation is that the IgG4 antibody might be induced by autoantigen on podocyte rather than PLA2R, for example, recently discovered THSD7A, which antibody has also been confirmed to be dominant IgG4 subclass [32]. The latter situation also appeared in 3 patients reported by Hoxha and colleagues [15]. How to explain the result? Perhaps, the dominant IgG subclass of PLA2R antibody in these patients was not IgG4. Several years ago Hofstra and colleagues [10] tested IgG subclasses of serum anti-PLA2R antibody in patient with PMN and revealed that in 5–7% cases the anti-PLA2R antibody was not IgG4 subclass. Hofstra’s finding supports our presumption.

In 2013 Huang and colleagues [38] found that IgG1 was the dominant IgG subclass in glomerular capillary deposits in early stage (stage 1) of PMN, but in all later stages IgG4 became dominant. They considered that there was an IgG subclass switching during disease progression of PMN. We utilized the same analytical method to observe whether a similar phenomenon also existed in our patients, but our results did not support Huang’s observation. We did not find any change of predominant/ codominant IgG subclass from early to late stages of PMN.

Local activation of complement cascade is prerequisite for the development of PMN. However, It has be known that IgG4 antibody is unable to activate complement system via classical pathway [2,6,39], then how the complement system is activated in this situation? One explanation is that IgG4 may activate complement cascade via the mannose-lectin pathway [1–4,40]. Actually many years ago mannose binding lectin had been identified in the glomeruli in a high proportion of PMN patients [41]. Another possible explanation is that the IgG1, IgG2 or IgG3 subclass, which is accompanied with dominant IgG4 in glomerular deposits, may be responsible for activation of complement system via classical pathway [1–3,40]. In this study, among the 167 PMN patients with predominant/codominant IgG4 deposition in glomeruli, 70 had no deposition of any other IgG subclass, and 97 had co-deposition of one to three other IgG subclasses. The pathogenesis of PMN in these two different situations might be explained by the above two assumptions respectively.

The studies of PLA2R and IgG subclass in glomerular deposits in the patients with SMN are relatively limited in published articles, and most of them focus on the LN-MN [4]. In contrast with PMN, there was no glomerular PLA2R deposition in LN-MN [14–16,22]. In addition, IgG1, IgG2 and/or IgG3, rather than IgG4 subclass, tended to be more dominant in glomerular deposits of LN-MN [6,42,43]. The results of our study are consistent with the above data. Of 40 patients with LN-MN, nobody had PLA2R and predominant/codominant IgG4 deposition in glomeruli.

Among the articles published before 2015, we only found total 6 patients with HBV-MN who were inspected PLA2R in glomerular deposits [14,15,22]. Of them, 2 cases showed positive result [14]. In addition, there were total 27 patients with HBV-MN who were tested circulating anti-PLA2R antibody [14,15,18–21]. Of them, 3 cases were found to have this antibody [14,19,21]. How to explain glomerular PLA2R deposition and circulating anti-PLA2R antibody appeared in HBV-MN patients? One possibility is that PMN was misdiagnosed as HBV-MN because of no strict staining quality control during detecting glomerular deposition of HBV antigens and/or loose HBV-MN diagnostic criteria. In most of published articles, the diagnostic criteria of HBV-MN were not shown [14,15,18,19,22]. To avoid this misdiagnosis, in the present study we applied two kinds of antibodies from different sources and double staining techniques of immunofluorescence and immunohistochemistry to check HBV antigens in glomeruli, and strict criteria to diagnose HBV-MN. However, we still found that PLA2R and predominant/codominant IgG4 simultaneously appeared in the glomeruli of 2 patients with HBV-MN. In this situation, a possible explanation for the result is that there may be a coincidental occurrence of PMN and HBV-MN [1]. In addition, we have also noticed the recently published article by Hao and his colleagues[44], in which 64% (25/39) Chinese patients with HBV-MN exhibited positive PLA2R in glomerular deposits and among them 6 patients whose serum samples were available all showed positive serum PLA2R antibody. In the similar studies conducted in China, our study showed the positive rate of glomerular PLA2R deposition in HBV-MN patients was 7.7% (2/26), and Qiu’s study showed the positive rate of serum PLA2R antibody in HBV-MN patients was 6.3% (1/16) [21]. Why is there so big discrepancy between Hao' research results and our as well as Qiu’s research results? It is not clear and it might require large sample studies conducted by multiple nephrology centers in China to clarify.

In the present study there are 2 patients with M-MN who had glomerular PLA2R and dominant IgG4 deposition. One patient suffered from metastatic prostate carcinoma and his M-MN got spontaneous partial remission in spite of the persistent presence of carcinoma. Another patient suffered from colonic carcinoma and her M-MN got complete remission following treatment with cyclosporine A before carcinoma resection. In previous reports, of 22 M-MN patients who were inspected glomerular PLA2R deposition, 4 showed positive results. Among these 4 patients, 2 simultaneously had dominant IgG4 deposition in glomeruli, but the remaining 2 patients were not checked the glomeruli deposition of IgG subclasses [14–17,22]. In addition, of 18 M-MN patients who were examined glomerular deposition of IgG subclasses, 5 had predominant/codominant IgG4 deposition, but were not inspected glomerular PLA2R deposition [27,28]. So, it is possible that in a few patients with M-MN there is glomerular PLA2R and/or dominant IgG4 deposition. One possible interpretation for this phenomenon is that this is a coincidence of PMN and malignancy, and therefore PMN was misdiagnosed as M-MN [1,2,21,22]. So far, there are no conformably accepted diagnostic criteria of M-MN, so it is easy to cause diagnostic confusion. Another possible interpretation is that an aberrant expression of PLA2R nephritogenic epitopes by the tumor cells leads to autoimmunity resembling that seeing in PMN [4,22]. In this study remission of M-MN is unrelated with carcinoma removing, which might be more in favor of the former hypothesis.

There is only one patient with IgG4-MN in our study. This patient had IgG4 but no PLA2R in glomerular deposits. The result is consistent with previous observation that IgG4-MN was not associated with glomerular PLA2R deposition as well as circulating anti-PLA2R antibodies [2,45–48]. The manifestation that is different from PMN suggests that there is different pathogenesis between these two diseases, and the PLA2R-related assays can help to differentiate these two diseases.

In conclusion, our study, that surveyed a large series of Chinese patients with PMN, LN-MN and HBV-MN, showed that glomerular PLA2R and dominant IgG4 deposition is frequently observed in the patients with PMN. In contrast, LN-MN and most of HBV-MN patients usually have no such PLA2R and IgG4 deposition. Therefore, detection of PLA2R and IgG subclasses in glomerular deposition may have an important value to distinguish between PMN and above-mentioned SMN.

## Supporting Information

S1 Table This is the original data of patients with MN. (XLSX)
